# Implementation of supply management strategies by the pharmacy service in a general hospital during the COVID-19 pandemic

**DOI:** 10.1016/j.rcsop.2022.100161

**Published:** 2022-07-26

**Authors:** Villalobos-Madriz Jorge, Zavaleta-Monestel Esteban, Serrano-Arias Bruno, Hernández-Fallas Yeralin, Diaz-Madriz Jose Pablo

**Affiliations:** a Department of Pharmacy Services, Hospital Clínica Bíblica, San José 10104, Costa Rica; b School of Pharmacy, Universidad de Ciencias Médicas, San José 10108, Costa Rica

**Keywords:** Supply management, Inventories, Hospital distribution systems, Pharmacy service, Hospital

## Abstract

**Objective:**

Given the global uncertainty faced due to the Covid-19 pandemic, health services were forced to adjust inventory management and purchase projections. This publication aims to describe the strategies taken and their impact on the supply chain indicators by the pharmacy service in the management of drug purchases during the pandemic to expose the importance of pharmacist in charge of the supply chain.

**Methods:**

This observational study describes the drug purchasing system in a general hospital and the strategies used to manage drug supply. The actions proposed by the pharmacy department are listed chronologically related to inventory issues and purchasing decisions. The accuracy of the purchase forecast was evaluated by calculating indicators such as the mean absolute standard deviation (MAD) and the variance of the forecast error (MSE). Inventory days and inventory turnover indicators were also compared in pre-pandemic and pre-pandemic periods.

**Findings:**

In general, the forecast error given by MAD and MSE tended to decrease. Specifically, from the 82 drug categories, during the pandemic period, this indicator decreased in 72 (88%), increased in 9 (11%), and remained the same in only 1 (1%) of the categories. In financial terms, comparing the 2018–2019 and 2020–2021 periods, a favorable result was obtained when evaluating the inventory turnover indicators, which decreased by 0.01 points and the days of inventory increased on average by two days.

**Conclusions:**

The implementation and use of these indicators prevented drug shortages, reducing inventory forecast errors. A pharmacist with knowledge in inventory management allows managing a process of continuous improvement and tactics for efficient inventory management without neglecting the benefit to patients or the economic profitability of the service. There were limitations since digital operating systems do not generate centralized or organized data for this type of analysis.

## 1 Introduction

On March 6, 2020, the first case of the virus was confirmed in Costa Rica and 10 days later, the country's health authorities declared a national state of emergency in response to the COVID-19 disease. During the month of April of that same year, the Costa Rican authorities generated important closures due to the uncertainty of the virus impact and the news that came from Europe.^1^^,^^2^ The limited information about the treatments available to treat the virus, and the sanitary restrictions, caused a significant impact, not only on the projections made by hospitals for the purchase of medicines within its catalog, but also on new acquisitions to respond to the drug needs that arose with the advance in the number of cases.^1^^,^3., 4., 5.

The COVID-19 pandemic led to the emergence of a new type of drug shortage that is attributed to the increase on its demand.^6^^,^^7^ Anticipating drug shortages by accumulation of stock, increase in number of patients, prolongation of hospital stays, and the use of medicines based on non-scientific information or social networks, were the main reasons for the increase in the demand for medicines due to the pandemic.8., 9., 10., 11.

Central America does not have published studies related to drug shortages and their impact on public health. These occur without warning, which is why robust safety stocks are ideal.^6^ The pharmacy service leads the proposals and decision-making in planning processes and strategies in the hospital's purchasing area, and so these are the professionals that participate in the design of treatment guides, protocols on alternative therapeutic options, management of therapies and prioritization of use of medications.12., 13., 14., 15., 16.

Pharmacists play an active role in formulating and managing special approvals for certain medications and conducting drug demand analysis to identify therapies of interest in the context of the pandemic.^12^^,^^15^ To measure the impact of these efforts, economic performance indicators related to inventory control and purchase projections are used; demonstrating the balance that a hospital must have in financial matters to ensure its continuity.^13^^,^^17^^,^^18^

This publication aims to describe the strategies and their impact on the supply chain indicators by the pharmacy service in the management of drug purchases during the covid-19 pandemic, to expose the importance of the pharmacist in charge of the supply chain.

## 2 Ethical considerations

This research is exempt from any ethics board approval because, according to the laws of the country and the National Health Research Council, it does not belong in the category of health research due to its economic nature.

## 3 Material and methods

This observational describes the drug purchasing system in a general hospital and the strategies used to manage the drug supply. The actions proposed by the pharmacy department are listed chronologically related to inventory issues and purchasing decisions. Subsequently, the actions taken within a period prior to the pandemic and in the middle of the pandemic are compared.^19^

As inclusion criteria for the analyzed data, information on tenders, scheduled product lines, direct purchases and actions taken was used, in the period prior to the pandemic comprised of the years 2018–2019 and during the pandemic between 2020 and 2022. Product lines that were not cataloged as medicines or that did not cover the periods studied were excluded. Key indicators were used for inventory management such as inventory turnover, inventory rotation, days of inventory, forecast error of bids and availability of medicines to describe the impact of the steps taken in the periods studied.

To evaluate the accuracy of the purchase forecast, the forecast error was calculated using the mean absolute standard deviation (MAD) which includes the average of absolute deviations in the period studied and expresses the absolute error as a percentage of demand. On the other hand, the mean square error (MSE), an indicator that contemplates the variance of the prognostic error, was compared in the same way. Finally, the projected data of purchases in the medicines tenders were compared with respect to the purchases made and the final rotation of the medicines by categories according to the therapeutic classification, expressing the percentage error that allows measuring the deviation of the prognosis with respect to the variations of the demand for drug lines.^20^^,^^21^

## 4 Results

### 4.1 Strategies used to manage the supply of medicines during the crisis

The Clínica Biblica's hospital drug purchasing system is based on a centralized model in its own drug distribution center, where an average of 60% of these items are acquired through the bidding modality, and the projections are made through the Forecast pro® program. Projections are based on historical sales data. The remaining 30% is purchased through direct purchases projected for a month of supply. There is a catalog of around 2200 items, the hospital does not limit prescription to medical staff, and new products are acquired for the hospital catalog every month. Therefore, taking actions in the acquisition of medicines during the pandemic was divided into two paths; the main one that consisted in ensuring the necessary inventory to treat hospitalized patients and, on the other hand, to follow up the behavior in the sales of pharmacies directed to the external consultation to avoid over-inventory, avoiding the impact of mobility restrictions and disease patterns.

The hospital plans purchase with four auctions per year scheduled in January, April, July, and October, each covering 3 months of rotation. However, due to the global situation, a change was made in the bidding schedule and in the number of product lines tendered, which is shown in Table 1.

**Table 1:** Bidding dates and number of product lines scheduled for purchase.

Year	2018	2019	2020	2021
Bid 1	January (1345)	January (1275)	January (1502)	January (1229)
Bid 2	April (1088)	April (1439)	August (262)	April (1272)
Bid 3	July (1465)	July (1390)	September (951)	July (1111)
Bid 4	October (1337)	October (1269)	October (449)	October (1260)
Bid 5	–	–	November (1189)	–
Total	5235	5373	4353	4872

Initially, the pharmacy department generated a list of essential medications to equip an Intensive Care Unit (ICU) focused on the treatment of patients with respiratory failure, so together with the chief physician of said department they define a list of 15 active ingredients vital (for example, sedatives such as Propofol and Midazolam or anticoagulants such as Enoxaparin). To measure the volume, the reorganization plans of the hospital in the emergency area are analyzed, and the establishment of 20 exclusive beds for the care of patients with COVID-19. Purchases were then projected according to the consumption calculated for 100% occupancy with full doses calculated in patients with an average weight of 80 k. This analysis was transmitted to the purchasing and procurement department, to measure medication availability, delivery times and brands available in the market.

**Fig. 1:**
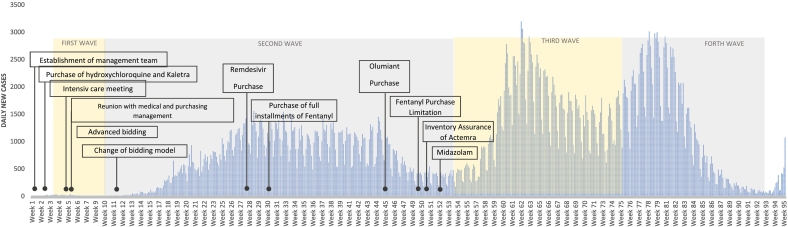
Actions taken in the hospital related to the acquisition of medicines.

In parallel, the supply chain coordinator pharmacist together with the clinical pharmacist analyzed the new treatment protocols used internationally to combat the disease. Initially, a supply of Hydroxychloroquine and Lopinavir/Ritonavir was guaranteed. At the request of infectology and critical care, Remdesivir was purchased, which was particularly complex because in Costa Rica said drug does not have a sanitary registration, therefore, each purchase a procedure had to be conducted with the Ministry of Health per patient for its importation; Baricitinib was acquired in the same way. In the case of Tocilizumab, we worked directly with the manufacturer, ensuring inventory for chronic patients registered in the pharmaceutical care program for the entire year 2021 and to supply hospitalized patients, which is reflected chronologically in Fig. 1 according to with the behavior of the pandemic in the country.

In the case of medicines defined as vital, the strategy was based on direct communication with distributors in the country every 15 days to measure inventory levels and ensure purchases for 3 months based on consumption. Fentanyl was purchased for the full quota approved by the Ministry of Health for the hospital, even though consumption did not reach 50% of it per month; this ensured the inventory of said drug since in 2021 the Ministry restricted the sale of the drug to the private sector to half the purchase quota per hospital for several months, due to the increase in consumption in social security. Access to Midazolam was reduced, since the brand available in the country for the private sector reported shortages; however, previous negotiations with a local manufacturing laboratory ensured the manufacture of a production batch for the hospital, acquiring enough quantity for 1 year, however, it served to contain the increase in consumption of more than 150% in the first months of 2021.

As shown in Table 2, the forecast error was calculated using MAD and MSE estimated for two years pre-pandemic 2018 and 2019; and for 2020 and 2021. The catalog of tendered medicines was divided according to Anatomical Therapeutic Chemical Classification System (ATC) category, resulting in 82 categories, based on the MAD, during the pandemic period this indicator decreased by 72 (88%), increased by 9 (11%) and it remained the same in only 1 (1%) of the categories. Among the specific drugs that directly affected the indicator, where the prognosis was affected during the pandemic, were antiprotozoals and antigout drugs, which include drugs such as ivermectin or colchicine.

**Table 2:** Indicators of forecast error in tenders in the pre-pandemic period (2018–2019) and during the pandemic (2020−2021).

Indicator	Before Pandemic	During Pandemic
MAD	127,20	78,80
MSE	333,80	318,3
Inventory turnover	0,57	0,56
Inventory days	52,00	54,00

MAD: mean absolute standard deviation. MSE: mean square error.

In financial terms, the results included in Table 2, the previously defined time periods can be compared with two indicators: inventory turns and days of inventory. In the first indicator for pharmacies, it decreased by 0.01 points. In the case of days of inventory, on average they increased by two days, when comparing the periods 2018–2019 and 2020–2021.

## 5 Discussion

The actions taken involved an increase in the inventories of medicines and supplies that will be used in the care of the pandemic, however, the mobility restrictions, the change in the pattern of circulating diseases in the population due to hygiene measures, as well as the economic deterioration of the population, reduced people's purchasing power, and this had repercussions on the sales of pharmacies.^1^^,^^3^

This situation caused measures to be taken to contain purchases of the drug categories in which sales were being reduced, as well as a change in the planning of purchases, since the projections are based on previous consumption, and with historical data that did not reflect the moment that the country was experiencing, the three-month projections were risky and the forecast error could be increased, bringing consequences in the accumulation of unnecessary inventory and reducing the available cash flow for the purchase of other necessary inputs.^1^^,^^3^

As shown in Table 1, a change was made in the programming of the auctions and in the number of lines auctioned. The main changes were made in 2020 as mobility limitations, imposed restrictions, cancellation of surgeries, office closures and the general uncertainty landscape reduced medication consumption by 60%; therefore, inventory management had to be agile to support patients and maintain the expected financial indicators; reducing the lines to bid as well as generating more frequent bids are strategies used to mitigate variability and reduce forecast error. However, a change had to be made in the programming of the tenders and in the number of lines tendered, which is shown in the Table 2.

In contrast, in the year 2021 the increase in hospitalization for patients sick with COVID-19; raised the average hospital stay by 45% in intermediate care and 140% in the intensive care unit compared to 2019.^1^^,^^3^ This generated increases in the consumption of medicines by 45%.^7^^,^^10^ These fluctuations caused measures to be taken to drastically vary the products that initially would be requested to remove products of greater use at the hospital level and negotiate the purchase directly with the supplier. Given this situation, the acquisition and purchase actions listed in Fig. 1 were taken, where the actions that were supposed to prevent a negative impact on the inventory indicators can be seen chronologically.

Analyzing the data presented in Table 2, when calculating the forecast error using MAD and the MSE, it was appreciated that these 2 indicators tended to reduce. As these results include the absolute error or, alternatively, the deviation of the inventory predictions, a decreased value represents that the error between the pre-pandemic measurements and during the pandemic was reduced. The foregoing shows that the actions described to mitigate inventory deficiencies and errors influenced these inventory accuracy markers.^20^

Considering the turn indicators and days of inventory also shown in Table 2, the first indicator for pharmacies had a minimal reduction, this interprets that the reinforcement of the lines to avoid shortages and the adaptation of the direct purchase model had already made an effect. The turnover of inventory decreased and did not increase as it would have happened in the event of a shortage of products. On the other hand, the days of inventory went from 52 to 54, which reflects that the inventory, having been reinforced, prolonged its existence by 2 days on average in all lines. Both numbers indicate that the changes in the purchasing system, as well as the extraordinary purchases to prepare for the pandemic, did not affect the financial results defined by the hospital.

The implemented strategies, constant monitoring, and communication with the medical management of the hospital made it possible to maintain a continuous stock of the medicines that were considered essential, and so no shortages of any medicine on the list of essential medicines were reported, and a quick response was given to the need for new products to combat the pandemic.

It should be noted that throughout the study there were limitations in terms of reports and that the digital operating systems or platforms do not generate data in an articulated manner and the management and processing of the information generated was conducted rudimentarily since the information is not centralized nor organized in an efficient way for this type of analysis.

## 6 Conclusion

A hospital's supply chain can be affected by economic and health factors worldwide. In the context of the global health emergency, establishing an inventory strategy that supports constant shifts and variations is vital for the provision of quality services. Pharmacy coordination is essential to have the necessary medications at the right time; the joint work of the clinical division with hospital acquisitions service allows not only to have up-to-date information, but also to plan, and improve response times. Measurement of inventory accuracy by means of the indicators discussed is a useful tool for prognostic quality supply chain management as this determines the quality of inventory which translates into the quantity and variety of stock for service needs. This is relevant since it makes it possible to manage a continuous improvement process and allows the design of tactics for efficient inventory management without neglecting the benefit to patients nor the economic profitability of the service. The actions taken and the indicators used not only allowed there to be no shortages, but also kept the financial indicators in line with the goal, and reduced forecast errors by giving a more prominent role in the purchasing system to the pharmacists in charge of inventory management.

## Funding

This research did not receive any specific grant from funding agencies in the public, commercial, or not-for-profit sectors.

## Declaration of Competing Interest

The authors declare that they have no known competing financial interests or personal relationships that could have appeared to influence the work reported in this paper.
